# Prevalence of Neutralizing Antibodies to Canine Distemper Virus and Response to Vaccination in Client-Owned Adult Healthy Dogs

**DOI:** 10.3390/v13050945

**Published:** 2021-05-20

**Authors:** Michèle Bergmann, Monika Freisl, Yury Zablotski, Md Anik Ashfaq Khan, Stephanie Speck, Uwe Truyen, Katrin Hartmann

**Affiliations:** 1 Clinic of Small Animal Medicine, LMU Munich, Veterinaerstrasse 13, 80539 Munich, Germany; m.freisl@medizinische-kleintierklinik.de (M.F.); Y.Zablotski@med.vetmed.uni-muenchen.de (Y.Z.); hartmann@lmu.de (K.H.); 2 Institute of Animal Hygiene and Veterinary Public Health, University of Leipzig, An den Tierkliniken 1, 04103 Leipzig, Germany; anik.ashfaq@gmail.com (M.A.A.K.); stephanie@speck-kaysser.de (S.S.); truyen@vetmed.uni-leipzig.de (U.T.)

**Keywords:** CDV, vaccine, antibody titer, virus neutralization, protection

## Abstract

Re-vaccinations against canine distemper virus (CDV) are commonly performed in 3-year intervals. The study’s aims were to determine anti-CDV antibodies in healthy adult dogs within 28 days of vaccination against CDV, and to evaluate factors associated with the presence of pre-vaccination antibodies and with the antibody response to vaccination. Ninety-seven dogs, not vaccinated within 1 year before enrollment, were vaccinated with a modified live CDV vaccine. A measurement of the antibodies was performed before vaccination (day 0), on day 7, and 28 after the vaccination by virus neutralization. A response to vaccination was defined as a ≥4-fold titer increase by day 28. Fisher’s exact test was used to determine factors associated with a lack of antibodies and vaccination response. In total, 94.8% of the dogs (92/97; CI 95%: 88.2–98.1) had antibodies (≥10) prior to vaccination. A response to vaccination was not observed in any dog. Five dogs were considered humoral non-responders; these dogs neither had detectable antibodies before, nor developed antibodies after vaccination. Young age (<2 years) was significantly associated with a lack of pre-vaccination antibodies (*p* = 0.018; OR: 26.825; 95% CI: 1.216–1763.417). In conclusion, necessity of re-vaccination in adult healthy dogs should be debated and regular vaccinations should be replaced by antibody detection.

## 1. Introduction

Distemper is a highly contagious infectious and often fatal disease. Canine distemper virus (CDV) is closely related to measles virus (MV). Vaccination has successfully reduced the number of clinical distemper cases [1], similarly to measles case numbers that have significantly decreased since the introduction of the measles vaccination [2], although an increasing number of measles cases has been observed in Europe in recent years [3]. The prevalence of distemper in dogs also has significantly been reduced since vaccination is available in veterinary medicine, but the risk of spreading CDV through dogs with an incomplete vaccination history, especially those imported from Eastern European countries, is still present [4]. Thus, all dogs should be protected against CDV infection at any time [5].

Similar to the protective situation in measles, the presence of antibodies against CDV in adult dogs, acquired through previous vaccination or exposure to field virus, predicts immunity against the disease [6,7,8]. There is consensus that any antibody titer (independent of the magnitude) detectable in an adult dog indicates protection, because the presence of antibodies is indicative of the presence of memory cells that can rapidly boost the dog’s antibody response in the event of infection [9].

Regular distemper re-vaccinations are recommended in veterinary medicine in at least 3-year intervals, although anti-CDV antibodies were present up to 9 years after modified live virus (MLV) vaccination in experimental virus-free conditions [7], and 4–14 years after vaccination in field conditions [7,10]. According to previous studies from Europe, Australia and the United States, between 72% [11] and 98% [12,13,14] of adult client-owned dogs have anti-CDV antibodies. 

Although rarely, vaccination against CDV can cause vaccine-associated adverse events (VAAEs), such as anaphylaxis [15] or neurological symptoms [16]. In a study that included a total of 85 dogs with allergic reactions within 24 h after vaccination, 28/83 dogs (33.7%) had previously been vaccinated with a combination vaccine against CDV, canine parvovirus (CPV) and canine adenovirus-2 (CAV-2) [17]. Therefore, a benefit–risk analysis should be performed prior to any vaccination [5]. In studies on CPV, animals with pre-existing anti-CPV antibodies either showed no increase in antibody titer or even a decline after MLV vaccination. Presumably, this was caused by pre-existing antibodies binding to the virus before stimulation of the immune system [18,19]. Corresponding data on CDV vaccination, however, are limited. One study evaluated the antibody response to modified live CDV vaccination in client-owned dogs that were presented for regular re-vaccination; only 12.4% of the dogs had a ≥4-fold titer increase [13]. So far, no study investigated whether specific factors are associated with the response to re-vaccination, but this knowledge would be helpful to improve CDV vaccination management.

The study’s aims were to evaluate (1) the prevalence of anti-CDV antibodies in adult dogs presented to a yearly health check appointment using virus neutralization (VN), (2) factors associated with the presence of pre-vaccination antibodies, (3) antibody response to vaccination with a MLV against CDV within a period of 28 days after vaccination, and (4) factors associated with the response to the vaccination.

## 2. Materials and Methods

### 2.1. Study Population

The protocol of this prospective study was accepted by the ethical committee of the LMU Munich (reference number: 55.2-1-54-2532.3-61-11). Ninety-seven dogs that were presented either to the Clinic of Small Animal Medicine, LMU Munich, a private practice in Southern Germany, or to a charity organization were included. 

Dogs were included if they (1) were at least 1 year of age, (2) had not received a CDV vaccination within the last 12 months, and (3) had an unremarkable disease history and physical examination at the time of presentation. Dogs were excluded if immunosuppressive drugs had been administered within the previous 4 weeks or if they had received serum preparations containing anti-CDV antibodies within the previous 12 months. Table 1 shows the characteristics of the dogs. 

### 2.2. Study Protocol

All dogs were vaccinated on day 0 with a MLV vaccine against canine distemper (strain Onderstepoort 10^4.0–6.0^ cell culture infective dose 50% (CCID_50_)) subcutaneously on the left lateral abdomen; the vaccine also contained CAV-2 and CPV (Nobivac^®^, MSD), which were not the subject of this study. Serum from the dogs was used for detection of pre-vaccination anti-CDV antibodies (on day 0) and anti-CDV antibodies after vaccination (on days 7 and 28). 

Various information about the dogs (signalment, origin, environment, housing conditions, daily contact to other dogs, travel and vaccination history) were collected from the owners (Table 1). Health status of the dogs was examined on days 0, 7, and 28. Owners had to report if VAAEs occurred during the study course.

Of all included dogs, 19.6% (19/97) had previously received a complete vaccination series against CDV. Dogs were considered to be completely vaccinated if they had received a completed primary vaccination series (a vaccination at least at the age of 16 weeks and 11–13 months later) and regular re-vaccinations at least every 3 years [5,20]. 

### 2.3. Detection of CDV Antibodies by VN

Sera of all dogs were heat-inactivated at 56 °C for 30 min and aliquots were stored at −20 °C. One hundred µL of each serum were pre-diluted (1:5) in phosphate-buffered saline (pH 7.2) and further serially diluted at steps of 1:2. Each dilution was mixed with an equal volume of CDV isolate Ag219 (200 median tissue culture infective dose per 0.1 mL), and incubated at 37 °C for 60 min. Subsequently, Vero-SLAM cells seeded in 96-well microtiter plates were inoculated with 100 μL of these serum/virus mixtures [21]. Plates were incubated for 5 days at 37 °C, 5% CO_2_. The positive control serum was obtained from a private-owned vaccinated dog. A titer <10 was considered negative [6,7]. A 4-fold titer increase, at least, by day 28 compared to day 0 was regarded as antibody response to vaccination. For comparison, VN against CDV isolate Onderstepoort was additionally performed in 27 sera from 9 dogs.

### 2.4. Reverse Transcription (RT)-PCR and Sequencing of the Full-Length Canine Distemper Hemagglutinin (H) Gene

The 2 canine distemper virus isolates CDV AG219 (Munich) and CDV Onderstepoort (Hannover) used for VN were sequenced to determine their homology based on the hemagglutinin gene. In brief, RNA was extracted by using Qiagen viral RNA kits (Qiagen, Hilden, Germany) according to the manufacturer’s instruction. RT-PCR was performed by using QIAGEN OneStep kits and with primer pairs that target CDV H gene, H-F (5′-TTAGGGCTCAGGTAGTCCA-3′; residue position: 7057–7075), and H-R (5′-CTAAGKCCAATTGARATGTGT-3′; residue position: 8935–8915; K = G/T, R = A/G) by following a protocol as published before [22]. The expected 1879-nucleotide amplicon that contains the full-length H gene was sequenced (Eurofins, Ebersberg, Germany). Poor-quality nucleotides off the ends of the test CDV H sequences were trimmed to obtain the final length of 1687 and 1678 nucleotides for CDV AG219 (Munich) and CDV Onderstepoort (Hannover, Germany) isolates, respectively.

Accessions of CDV H gene sequence from 181 worldwide strains and 7 modified live vaccine strains were taken from a published pool of manually cured and stratified GenBank entries [2]. Retrieved sequences were aligned to the test CDV H gene sequences using MUSCLE with default parameters, and pairwise identities of the test sequences were calculated. Statistical selection for best-fit model of nucleotide substitution was carried out using jModelTest2 on XSEDE server (https://www.phylo.org/) (accessed on 16 January 2021), and maximum-likelihood topology for each model was chosen for base tree likelihood calculations. Based on Bayesian inference criterion (BIC), transversion model of nucleotide substitution was selected with consideration of gamma-distributed rate variation. Phylogenetic tree was thus inferred by using IQTree-CIPRES (accessed from Geneious Prime v2020.1.2) with 1000 bootstrap replicates.

### 2.5. Statistical Analysis

Statistical analysis was performed with VN antibody results using isolate AG219. SPSS version 22 (IBM Corporation, Armonk, USA) was used for statistical analysis. A power analysis was performed before the start of the study and revealed a required sample size of 87 dogs assuming an antibody prevalence of 94% at a significance level of 95% with a power of 90%. Assessment of factors associated with presence of pre-vaccination antibodies (Table 1) and with antibody response to vaccination was performed using Fisher’s exact test. Multivariate logistic regression analysis was planned for factors significant in univariate analysis. For all analyses, a *p*-value <0.05 was considered significant. 

## 3. Results

### 3.1. Sequences of Isolate CDV AG219 and CDV Onderstepoort

**Figure 1:**
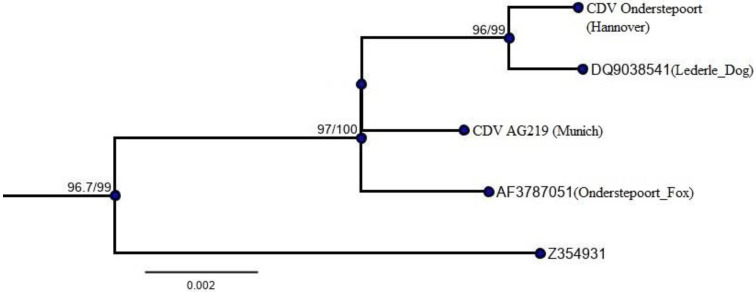
Phylogenetic relationship showing the position of test sequences in a clade of modified live vaccine strains within America-1 lineage. For key nodes, support values are labeled as bootstrap (BS)/posterior probability (PP).

The CDV AG219 (Munich) and CDV Onderstepoort (Hannover) isolates showed maximum pairwise identities of 99.585% and 99.702% to GenBank sequences of CDV Onderstepoort (location: South Africa; host: fox; accession: AF378705.1) and CDV Lederle (location: USA; host: dog; accession: DQ903854.1) strains, respectively (Table 2). The phylogenetic tree reconstructed for the obtained sequences showed that the genotypes of the test sequences based on H gene homology corresponded to the strains of the America-1 lineage [23]. Furthermore, both of the test isolates share common ancestral origin with the Onderstepoort strain (AF378705.1), with high branching support (Figure 1), and are unlikely to be originated from other worldwide lineages (Supplementary Figure S1). The obtained nucleotide sequences can be retrieved from https://doi.org/10.5281/zenodo.4562485 (accessed on 11 March 2021).

### 3.2. Comparison of CDV Antibodies in VN Using Isolate Ag219 and Onderstepoort

Antibodies against CDV AG219 (Munich) and CDV Onderstepoort were present in all of the 27 serum samples in which a comparison was performed. Twelve samples revealed the same identical titer and 15 samples differed, but only in 1 titer step (Table 3).

### 3.3. Pre-Vaccination Antibodies against Isolate CDV AG219

Overall, 94.8% of the dogs (92/97; CI 95%: 88.2–98.1) had pre-vaccination antibody titers ≥10 by day 0 (median titer: 80, range: <10–640; mean titer: 114 ± 141). Table 4 shows details of the five dogs without pre-vaccination antibodies on day 0. A young age was associated with a lack of pre-vaccination antibodies in univariate analysis (Table 1). The dogs <2 years of age were more likely to lack antibodies than the dogs between 2–≤ 9 years of age (*p* = 0.018; OR: 26.825; 95% CI: 1.216–1763.417). As no other characteristics were associated with the presence of pre-vaccination antibodies in univariate analysis, a multivariate analysis was not performed.

### 3.4. Titer Increase after Vaccination against Isolate CDV AG219

**Figure 2:**
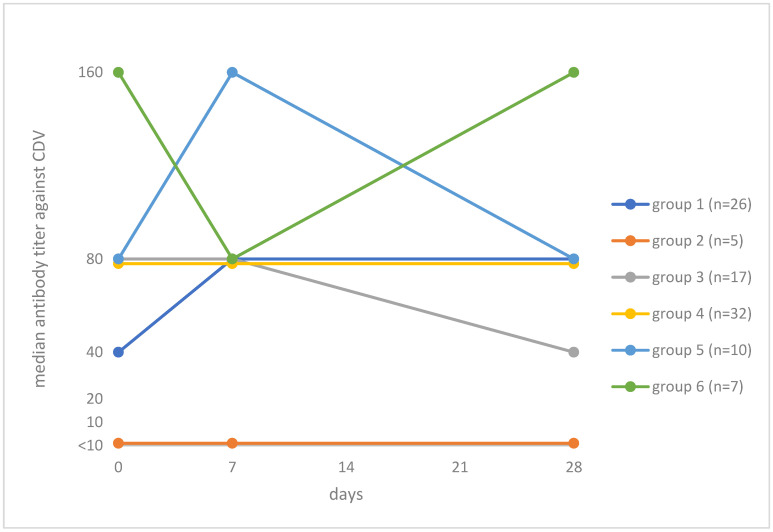
Grouping of dogs based on median antibody titers against canine distemper virus (CDV) on day 0 and changes in titer after vaccination against CDV in virus neutralization (VN) testing using isolate AG219. The antibody titers of the individual dogs are given in Table S1.

None of the dogs had a ≥4-fold titer increase after vaccination. A titer increase <4-fold (only one titer step) was observed in 26.8% (26/97; 95% CI: 19.0–36.4) of the dogs. The dogs were categorized according to their antibody response to vaccination (Figure 2). The dogs in group 1 (*n* = 26; 26.8%) had an antibody titer ≥10 on day 0 and an increase in titer by day 28. The dogs in group 2 (*n* = 5; 5.2%) were considered as humoral non-responders; these dogs had no antibodies (≥10) before vaccination and did not develop antibodies after vaccination either. The dogs in group 3 (*n* = 17; 17.5%) had an antibody titer ≥10 on day 0 and a decrease in titer by day 28. The dogs in group 4 (*n* = 32; 33.0%) had an antibody titer ≥10 on day 0 and no change in titer after vaccination. The dogs in group 5 (*n* = 10; 10.3%) had an antibody titer ≥10 on day 0, an increase in titer by day 7 and a decrease in titer by day 28. The dogs in group 6 (*n* = 7; 7.2%) had an antibody titer ≥10 on day 0, a decrease in titer by day 7 and an increase in titer by day 28. Table S1 shows the antibody titers of the individual dogs throughout the study.

- Group 1: Titer ≥ 10 on day 0 and subsequent increase in titer by day 28 (*n* = 26; 26.8%). Median titers: day 0, 40; day 7, 80; day 28, 80;
- Group 2: Titer < 10 pre- and titer < 10 post-vaccination (*n* = 5; 5.2%). Median titers on each day <10;
- Group 3: Titer ≥ 10 on day 0 and subsequent decrease in titer by day 28 (*n* = 17; 17.5%). Median titers: day 0, 80; day 7, 80; day 28, 40;
- Group 4: Titer ≥ 10 on day 0 and no change in titer after vaccination (*n* = 32; 33.0%). Median titers: day 0, 80; day 7, 80; day 28, 80;
- Group 5: Titer ≥ 10 on day 0, an increase in titer by day 7 and a decrease in titer by day 28 (*n* = 10; 10.3%). Median titers: day 0, 80; day 7, 160; day 28, 80;
- Group 6: Titer ≥ 10 on day 0, a decrease in titer by day 7 and an increase in titer by day 28 (*n* = 7; 7.2%). Median titers: day 0, 160; day 7, 80; day 28, 160.

### 3.5. Vaccine-Associated Adverse Events

Overall, 36.1% (35/97; 95% CI_:_ 27.2–46.0) of dogs showed at least one VAAE. The most common VAAEs included lethargy (23.7%; 23/97), inguinal or popliteal lymphadenopathy (19/97; 19.6%), or gastrointestinal signs (15/97; 15.5%). Injection-site reactions with local changes (swelling and pain) were only recorded in one dog. The occurrence of VAAEs was not significantly associated with a titer increase after vaccination.

## 4. Discussion

Similar to human measles, at least 90–95% of dogs should be immune against CDV in order to prevent outbreaks [24,25]. In the present study, 94.8% of all the dogs had neutralizing antibodies against the CDV isolate Ag219, which indicates an appropriate level of immunity. The isolate Ag219 belongs to the Onderstepoort/Lederle group; it is almost identical to the vaccine strain that was used to vaccinate the dogs in the present study (Figure 1). Thus, dogs developing antibodies after vaccination should have been detected with the assay used. The high number of dogs with pre-existing antibodies might be explained by the fact that all the dogs had been vaccinated in the past. After MLV vaccination against CDV, antibodies develop within 13–15 days [26] and persist for 2–14 years [6,7,9,10,11,27]. Interestingly, the overall antibody titers in the dogs from the present study were low (median antibody titer on day 0: 80). This is similar to the results of a study by Jozwik et al. (2004) who measured the antibodies in vaccinated dogs by an immunoperoxidase monolayer assay, also using a Lederle strain (mean antibody titer: 114). Lederle as well as Onderstepoort are egg-adapted strains in which virulent properties are reduced, and it is possible that vaccination with these strains induces only low levels of antibodies [28]. However, these strains provide sufficient protection since no CDV outbreaks (in vaccinated dogs) have been reported in countries in which only vaccines with Lederle or Onderstepoort strains are available. Besides vaccination, antibodies can be present due to previous exposure; CDV remains infectious in the environment for several days depending on the temperature [29,30]. A study in dogs originating from the same geographical region as the dogs in the present study failed to detect CDV in oral and nasal swabs from healthy dogs and dogs with canine infectious respiratory disease [31], but fecal shedding of paramyxovirus and thus environmental contamination was found in 9.3% of dogs with acute hemorrhagic diarrhea from the same region in another study [32]. 

In the present study, none of the dogs had a ≥4-fold titer increase after vaccination. It has to be discussed whether this was influenced by the use of the CDV isolate Ag219 in VN. Testing the sera of dogs against both of the CDV isolates (Ag219 and Onderstepoort) in the present study, however, revealed nearly identical antibody titers. Thus, false-negative VN results are less likely. In a study by Mitchell and coworkers (2012), in which client-owned dogs were re-vaccinated with a combined MLV vaccine against CDV, 12.4% of dogs had a ≥4-fold titer increase 7–14 days after vaccination. The most likely cause for the higher response rate might be the lower number of dogs with pre-vaccination antibodies (84.1%) in the study of Mitchell and coworkers, compared to 94.8% in the present study. The lower the pre-vaccination titer, the more likely MLV will replicate, and therefore stimulate an antibody response. 

In the present study, five dogs had no pre-vaccination antibodies, although all of these dogs had been vaccinated in the past. It is possible that these dogs were humoral non-responders. Humoral non-responding to vaccination might occur because the immune system fails to recognize the vaccine antigen [33]. Humoral non-responders have been reported in 0.02–1.0% of dogs [5,9,34], but this percentage has probably been underestimated. In humans, approximately 2–10% fail to develop antibodies after MLV vaccination against measles [35]. The immunological reasons for non-responding and the resultant consequences are largely unexplored. In human medicine, non-responding is well-known, especially after vaccination against hepatitis B virus, and several factors, e.g., genetic predisposition, chronic diseases, obesity and smoking, are considered to be responsible [35,36]. In dogs, breed-specific variations in the genes of the immune system coding for specific proteins that are necessary for antigen presentation to lymphocytes have been discussed as an important reason for humoral non-responding [37,38,39,40]. Such variations have not only been described for specific breeds, but also for dog populations from different geographic regions, which could explain why individual breeds or even breeding lines within a breed differ in their immune response to certain vaccinations [41]. Furthermore, variations in the circulating lymphocyte subpopulations [42], and polymorphisms in cytokine genes involved in the regulation of Th1/Th2 balance, such as interleukin-10 [43], could contribute to non-responding. Finally, primary immunodeficiency syndromes can be responsible for non-responding, such as canine leucocyte adhesion deficiency, e. g., in Setters [44,45,46,47]. It is unknown whether humoral non-responders have also impaired cellular immune responses, and thus generally a higher risk for acquiring disease. It could even be discussed whether these dogs have lower susceptibility to CDV infection or might lack receptors for CDV cell entry. A definitive answer on why the five dogs in the present study did not develop neutralizing antibodies could only be given through challenge experiments, which cannot be performed on privately owned dogs. The absence of neutralizing antibodies in previously vaccinated dogs did not result in susceptibility to disease on the CDV challenge [6]. Furthermore, humoral as well as cell-mediated immune responses determined by lymphocyte transformation tests could be detected in dogs after vaccination against canine distemper, indicating that cellular immunity plays an important role [48]. Further studies should be performed to determine whether humoral non-responders are protected against disease or not, and thus eventually should not be used for breeding. So far, and to the current state of knowledge, the authors recommend regular revaccinations in humoral non-responders with the aim to at least boost cellular immunity. 

One aim of the present study was to evaluate the association of different factors with the lack of pre-vaccination antibodies. A lack of previous vaccinations [27,49] or regular re-vaccinations [50] resulted in lower or missing CDV titers in previous studies, but the results differed and associations between vaccination history and lack of CDV antibodies could not be confirmed in other studies [11,13,49,51]. In the present study, many dogs had pre-vaccination antibodies, although most of them had not been fully vaccinated in the past according to current guidelines. This is in line with the results from Schultz et al. (2010) concluding that missing CDV re-vaccinations in adult dogs is not problematic if these dogs have been vaccinated at least once at a time when maternally derived antibodies (MDA) were no longer present. 

In the present study, the dogs <2 years had a higher probability of lacking pre-vaccination antibodies than the dogs between 2–≤ 9 years. Several previous studies found that a younger age resulted in lower levels of antibodies [49,52,53], although some studies showed the contrary [50,54]. It is generally assumed that older dogs were exposed to CDV for a longer period and therefore were more likely to have antibodies. Otherwise, MDA interference during the primary vaccination series in puppies might be a reason for vaccination failure and the subsequent lack of antibodies. The dogs >9 years were not more likely to lack antibodies compared to the dogs between 2–≤ 9 years in the present study, suggesting that older dogs do not need more frequent re-vaccinations. It is well known that ageing is linked to a decline in immune functions in humans [55]. In contrast to human medicine, susceptibility to infections seems not to be higher in older dogs, but it is unclear how ageing influences vaccination success. One study found that dogs failed to mount an adequate immune response to the rabies virus after a first vaccination at an advanced age [56]. However, in a study of Schultz and coworkers, CDV antibodies were present for at least 9 years after vaccination and dogs were resistant against challenge even after a single dose of an MLV vaccine [7]. There was only a small number of dogs aged <2 years in the present study cohort and young age was the only factor associated with a lack of antibodies. Therefore, although the difference between age groups was significant, it would be valuable to verify this difference using a larger number of young dogs. Except for the factor of “young age”, no other factor was significantly related with the lack of antibodies. It should, however, be noticed that the results of the statistical analysis are based on only a small number of dogs lacking pre-vaccination antibodies, and thus should be interpreted cautiously. This also was the main limitation of the study. However, the small number of dogs lacking pre-vaccination antibodies mimics the natural situation of dogs presented to veterinarians for revaccination.

## 5. Conclusions

Neutralizing pre-vaccination antibodies against CDV were found in almost all of the dogs in the present study, even in those that had been vaccinated >5 years before the beginning of the study. None of the dogs were successful in developing a ≥4-fold titer increase in neutralizing antibodies after re-vaccination. The necessity of re-vaccination in adult healthy dogs should therefore be debated and re-vaccination should be replaced by antibody detection. 

## Figures and Tables

**Table 1:** Characteristics of dogs and their association with presence of antibodies against canine distemper virus isolate AG219 in Fisher’s exact test.

Variable	Category	Number of Dogs Tested	Dogs with Pre- Vaccination Antibodies Against CDV ^1^	Univariate Analysis		
				*P* ^2^	Odds Ratio	95% CI ^3^
Age	<2 years	7/97	5/7	0.018	26.8	1.2–1763.4
	2 ≤ 9 years	76/97	75/76	Ref.value ^4^	n. a. ^5^	n. a. ^5^
	>9 years	14/97	12/14	0.062	11.9	0.6–742.6
Sex	Female	57/97	55/57	0.401	-	-
	Male	40/97	37/40			
Weight	<10 kg	16/97	15/16	0.511	-	-
	10–20 kg	23/97	23/23			
	20–30 kg	31/97	28/31			
	>30 kg	27/97	26/27			
Neutering status	Intact	47/97	44/47	0.671	-	-
	Neutered	50/97	48/50			
Origin	Breeder	33/97	32/33	0.641	-	-
	Private	23/97	21/23			
	Shelter	13/97	12/13			
	Humane society	28/97	27/28			
Environment	Urban	56/97	54/56	0.647	-	-
	Rural	41/97	38/41			
Lifestyle	Family	70/97	66/70	1.000	-	-
	Breeding	9/97	9/9			
	Farm	4/97	4/4			
	Utility	14/97	13/14			
History abroad	Yes	65/97	62/65	1.000	-	-
	No	32/97	30/32			
Housing conditions	Other dogs/cats	62/97	57/62	0.156	-	-
	No other dogs/cats	35/97	35/35			
Daily contact with other dogs	<2	20/97	20/20	0.079	-	-
	3–5	58/97	56/58			
	>5	19/97	16/19			
Time since last vaccination	1–3 years	77/97	73/77	0.203	-	-
	3–4 years	11/97	11/11			
	4–5 years	4/97	4/4			
	5–7 years	3/97	3/3			
	>7 years	2/97	1/2			
Complete vaccination series	Yes	19/97	18/19	1.000	-	-
	No	78/97	74/78			

^1^ CDV, canine distemper virus; ^2^ *P*, *p*-value; ^3^ CI, confidence interval; ^4^ Ref.value, reference value; ^5^ n. a., not applicable. Bold values indicate statistically significant differences.

**Table 2:** Nucleotide and amino acid identity features of the test isolates for H genes and proteins against modified live vaccine strains of Onderstepoort and Lederle.

	Nucleotide Mismatch/Percent Identity; Amino Acid Mismatch/Percent Identity	
	CDV Onderstepoort (Hannover)	CDV Onderstepoort (AF378705.1)
CDV AG219 (Munich)	9/99.404; 6/98.927	7/99.585; 3/99.466
CDV Lederle (DQ903854.1)	4/99.702; 3/99.463	11/99.394; 6/99.007

CDV, canine distemper virus.

**Table 3:** Comparison of the results of virus neutralization (VN) using isolate Ag219 and Onderstepoort.

Serum	Dog	Sample	VN isolate Ag219	VN isolate Onderstepoort
1	1	day 0	20	20
2	1	day 7	20	40
3	1	day 28	40	40
4	2	day 0	40	20
5	2	day 7	80	40
6	2	day 28	40	40
7	3	day 0	160	160
8	3	day 7	160	160
9	3	day 28	80	160
10	4	day 0	80	80
11	4	day 7	80	80
12	4	day 28	80	40
13	5	day 0	40	80
14	5	day 7	80	80
15	5	day 28	80	80
16	6	day 0	40	80
17	6	day 7	80	80
18	6	day 28	80	160
19	7	day 0	10	20
20	7	day 7	10	20
21	7	day 28	10	10
22	8	day 0	40	80
23	8	day 7	40	80
24	8	day 28	40	80
25	9	day 0	40	20
26	9	day 7	40	20
27	9	day 28	40	40

**Table 4:** Dogs without pre-vaccination antibodies against canine distemper virus isolate AG219 on day 0.

Dog	Signalment	Weight	Origin, Lifestyle	Environment	Daily Contact to Other Dogs	Previous Vaccinations	Time since Last Vaccination	Complete Vaccination Series *	CDV ^1^ Antibody Titer			VAAEs ^2^ after Vaccination
									Day 0	Day 7	Day 28	
1	Miniature Pinscher, 2 y ^3^, female, intact	4 kg ^4^	Private, family dog	Urban	>5	7 w ^5^ 15 w	1 y, 7 m ^6^	No	<10	<10	<10	No
2	Labrador, 7 y, female, neutered	26 kg	Private, farm dog	Rural	3–5	8 m 5 y 6 y	1 y	No	<10	<10	<10	Yes Lethargy day 0–7
3	Bavarian Mountain Hound, 2 y, male, intact	22 kg	Breeder, utility dog	Rural	>5	9 w 13 w 18 w 1 y	1 y, 2 m	Yes	<10	<10	<10	Yes Lethargy day 0–7
4	Mix, 1.5 y, male neutered	20 kg	Humane society, family dog	Urban	>5	16 w 20 w	1 y, 2 m	No	<10	<10	<10	No
5	Mix, 13 y, male neutered	43 kg	Shelter, family dog	Rural	3–5	5 m 6 m	12 y	No	<10	<10	<10	Yes Local reactions day 7–28

^1^ CDV, canine distemper virus; ^2^ VAAEs, vaccination adverse events; ^3^ y, years; ^4^ kg, kilogram; ^5^ w, weeks; ^6^ m, months. *A complete vaccination series against CDV was defined as follows: In puppies, vaccination was started at 6–8 weeks of age and boosters were given at 3–4 week intervals until at least 16 weeks of age. A booster vaccination had to be given 11–13 months later. In dogs ≥12 weeks, vaccination was considered complete if they had received two vaccinations in a 3–4 week interval with a final booster after 11–13 months; after the primary vaccination series, booster vaccinations were required at intervals of no greater than 3 years.

## Data Availability

The authors confirm that the datasets analyzed during the study are available from the first author or the corresponding author upon reasonable request.

## Supplementary Materials

The following are available online at https://www.mdpi.com/article/10.3390/v13050945/s1, Figure S1. Phylogenetic position of test sequences (shaded yellow) among the clades of different lineages, Table S1. Anti-canine distemper antibody titers in virus neutralization using isolate AG219 in 97 dogs.

## References

[1] Dabbagh A., Patel M.K., Gacic-Dobo M., Mulders M.N., Okwo-Bele J.M., Kretsinger K., Papania M.J., Rota P.A., Goodson J.L. (2017). Progress toward regional measles elimination—Worldwide, 2000–2016. MMWR Morb. Mortal. Wkly. Rep..

[2] World Health Organization Measles. https://who.int/news-room/fact-sheets/detail/measles.

[3] Koch A., Krönert C., Lotti T., Vojvodic A., Wollina U. (2019). Adult measles—Case reports of a highly contagious disease. Open Access Maced. J. Med. Sci..

[4] Willi B., Spiri A.M., Meli M.L., Grimm F., Beatrice L., Riond B., Bley T., Jordi R., Dennler M., Hofmann-Lehmann R. (2015). Clinical and molecular investigation of a canine distemper outbreak and vector-borne infections in a group of rescue dogs imported from Hungary to Switzerland. BMC Vet. Res..

[5] Day M.J., Karkare U., Schultz R.D., Squires R., Tsujimoto H. (2015). Recommendations on vaccination for Asian small animal practitioners: A report of the WSAVA Vaccination Guidelines Group. J. Small Anim. Pract..

[6] Jensen W.A., Totten J.S., Lappin M.R., Schultz R.D. (2015). Use of serologic tests to predict resistance to canine distemper virus-induced disease in vaccinated dogs. J. Vet. Diagn. Investig..

[7] Schultz R.D., Thiel B., Mukhtar E., Sharp P., Larson L.J. (2010). Age and long-term protective immunity in dogs and cats. J. Comp. Pathol..

[8] Plotkin S.A. (2010). Correlates of protection induced by vaccination. Clin. Vaccine Immunol..

[9] Schultz R.D. (2006). Duration of immunity for canine and feline vaccines: A review. Vet. Microbiol..

[10] Olson P., Finnsdottir H., Klingeborn B., Hedhammar A. (1997). Duration of antibodies elicited by canine distemper virus vaccinations in dogs. Vet. Rec..

[11] Böhm M., Thompson H., Weir A., Hasted A.M., Maxwell N.S., Herrtage M.E. (2004). Serum antibody titres to canine parvovirus, adenovirus and distemper virus in dogs in the UK which had not been vaccinated for at least three years. Vet. Rec..

[12] McCaw D.L., Thompson M., Tate D., Bonderer A., Chen Y.J. (1998). Serum distemper virus and parvovirus antibody titers among dogs brought to a veterinary hospital for revaccination. J. Am. Vet. Med. Assoc..

[13] Mitchell S.A., Zwijnenberg R.J., Huang J., Hodge A., Day M.J. (2012). Duration of serological response to canine parvovirus-type 2, canine distemper virus, canine adenovirus type 1 and canine parainfluenza virus in client-owned dogs in Australia. Aust. Vet. J..

[14] Twark L., Dodds W.J. (2000). Clinical use of serum parvovirus and distemper virus antibody titers for determining revaccination strategies in healthy dogs. J. Am. Vet. Med. Assoc..

[15] Moore G.E., Guptill L.F., Ward M.P., Glickman N.W., Faunt K.K., Lewis H.B., Glickman L.T. (2005). Adverse events diagnosed within three days of vaccine administration in dogs. J. Am. Vet. Med. Assoc..

[16] Cußler K., Schwedinger E. (2012). Pharmakovigilanz für Qualität, Wirksamkeit und Unbedenklichkeit von Tierarzneimitteln, Nebenwirkungen nach Impfung von Deutschen Pinschern. DTBL.

[17] Ohmori K., Sakaguchi M., Kaburagi Y., Maeda S., Masuda K., Ohno K., Tsujimoto H. (2005). Suspected allergic reactions after vaccination in 85 dogs in Japan. Vet. Rec..

[18] Bergmann M., Schwertler S., Reese S., Speck S., Truyen U., Hartmann K. (2018). Antibody response to feline panleukopenia virus vaccination in healthy adult cats. J. Feline Med. Surg..

[19] Riedl M., Truyen U., Reese S., Hartmann K. (2015). Prevalence of antibodies to canine parvovirus and reaction to vaccination in client-owned, healthy dogs. Vet. Rec..

[20] Welborn L.V., DeVries J.G., Ford R., Franklin R.T., Hurley K.F., McClure K.D., Paul M.A., Schultz R.D. (2011). 2011 AAHA canine vaccination guidelines. J. Am. Anim. Hosp. Assoc..

[21] Seki F., Ono N., Yamaguchi R., Yanagi Y. (2003). Efficient isolation of wild strains of canine distemper virus in vero cells expressing canine SLAM (CD150) and their adaptability to marmoset B95a cells. J. Virol..

[22] Liao P., Guo L., Wen Y., Yang Y., Cheng S. (2015). Phylogenetic features of hemagglutinin gene in canine distemper virus strains from different genetic lineages. Int. J. Clin. Exp. Med..

[23] Ke G.M., Ho C.H., Chiang M.J., Sanno-Duanda B., Chung C.S., Lin M.Y., Shi Y.Y., Yang M.H., Tyan Y.C., Liao P.C. (2015). Phylodynamic analysis of the canine distemper virus hemagglutinin gene. BMC Vet. Res..

[24] Pan American Health Organization (1999). Measles Eradication: Field Guide.

[25] Rikula U., Nuotio L., Sihvonen L. (2007). Vaccine coverage, herd immunity and occurrence of canine distemper from 1990–1996 in Finland. Vaccine.

[26] Litster A., Nichols J., Volpe A. (2012). Prevalence of positive antibody test results for canine parvovirus (CPV) and canine distemper virus (CDV) and response to modified live vaccination against CPV and CDV in dogs entering animal shelters. Vet. Microbiol..

[27] Jozwik A., Frymus T., Mizak B., Rzezutka A. (2004). Antibody titres against canine distemper virus in vaccinated and unvaccinated dogs. J. Vet. Med. B Infect. Dis. Vet. Public Health.

[28] Appel M.J.G., Gillespie J.H., Gard S., Hallauer C., Meyer K.F. (1972). Canine distemper virus. Virology Monographs 11: Canine Distemper Virus Marburg Virus.

[29] Martella V., Elia G., Buonavoglia C. (2008). Canine distemper virus. Vet. Clin. N. Am. Small Anim. Pract..

[30] Newbury S., Larson L.J., Schultz R., Miller L., Hurley K. (2009). Canine distemper virus. Infectious Disease Management in Animal Shelters.

[31] Schulz B.S., Kurz S., Weber K., Balzer H.J., Hartmann K. (2014). Detection of respiratory viruses and *Bordetella bronchiseptica* in dogs with acute respiratory tract infections. Vet. J..

[32] Schulz B.S., Strauch C., Mueller R.S., Eichhorn W., Hartmann K. (2008). Comparison of the prevalence of enteric viruses in healthy dogs and those with acute haemorrhagic diarrhoea by electron microscopy. J. Small Anim. Pract..

[33] Haralambieva I.C.H., Kennedy R.B., Ovsyannikova I.G., Whitaker J.A., Poland G.A. (2015). Variability in humoral immunity to measles vaccine: New Developments. Trends Mol. Med..

[34] Larson L.J., Schultz R.D. (2007). Three-year duration of immunity in dogs vaccinated with a canarypox-vectored recombinant canine distemper virus vaccine. Vet. Ther..

[35] Filippelli M., Lionetti E., Gennaro A., Lanzafame A., Arrigo T., Salpietro C., La Rosa M., Leonardi S. (2014). Hepatitis B vaccine by intradermal route in non responder patients: An update. World J. Gastroenterol..

[36] Lambkin R., Novelli P., Oxford J., Gelder C. (2004). Human genetics and responses to influenza vaccination: Clinical implications. Am. J. Pharm..

[37] Day M.J. (2007). Immune system development in the dog and cat. J. Comp. Pathol..

[38] Day M.J., Schultz R.D., Day M.J., Schultz R.D. (2014). Vaccination. Veterinary Immunology—Principles and Practice.

[39] Tizard I., Tizard I. (2018). Dendritic cells and antigen processing. Veterinary Immunology.

[40] Tizard I., Tizard I. (2018). The major histocompatibility complex. Veterinary Immunology.

[41] Kennedy L.J., Barnes A., Happ G.M., Quinnell R.J., Courtenay O., Carter S.D., Ollier W.E.R., Thomson W. (2002). Evidence for extensive DLA polymorphism in different dog populations. Tissue Antigens.

[42] Faldyna M., Leva L., Knotigova P., Toman M. (2001). Lymphocyte subsets in peripheral blood of dogs-a flow cytometric study. Vet. Immunol. Immunopathol..

[43] Kennedy L.J., Lunt M., Barnes A., McElhinney L., Fooks A.R., Baxter D.N., Ollier W.E.R. (2007). Factors influencing the antibody response of dogs vaccinated against rabies. Vaccine.

[44] Debenham S.L., Millington A., Kijast J., Andersson L., Binns M. (2002). Canine leucocyte adhesion deficiency in Irish Red and White Setters. J. Small Anim. Pract..

[45] Foureman P., Whiteley M., Giger U. (2002). Canine leukocyte adhesion deficiency: Presence of the Cys36Ser beta-2 integrin mutation in an affected US Irish Setter cross-breed dog and in US Irish Red and White Setters. J. Vet. Intern. Med..

[46] Kijas J.M., Bauer T.R., Gafvert S., Marklund S., Trowald-Wigh G., Johannisson A., Hedhammar A., Binns M., Juneja R.K., Hickstein D.D. (1999). A missense mutation in the beta-2 integrin gene (ITGB2) causes canine leukocyte adhesion deficiency. Genomics.

[47] Pfeiffer I., Brenig B. (2005). Frequency of the canine leucocyte adhesion deficiency (CLAD) mutation among Irish Red Setters in Germany. J. Anim. Breed. Genet..

[48] Gerber J.D., Marron A.E. (1976). Cell-mediated immunity and age at vaccination associated with measles inoculation and protection of dogs against canine distemper. Am. J. Vet. Res..

[49] Rikula U., Nuotio L., Sihvonen L. (2000). Canine distemper virus neutralising antibodies in vaccinated dogs. Vet. Rec..

[50] Schoder D., Benetka V., Sommerfeld-Stur I., Kopf N., Weissenbacher E., Pallan C., Walk K., Möstl K. (2006). Untersuchungen zum Antikörper-Status gegen Hundestaupe-Virus und canines Parvovirus-2 bei Hunden in Niederösterreich und Wien nach unterschiedlichen Impfintervallen. Vet. Med. Austria/Wien. Tierärztl. Mschr..

[51] Olson P., Klingeborn B., Hedhammar A. (1988). Serum antibody response to canine parvovirus, canine adenovirus-1, and canine distemper virus in dogs with known status of immunization: Study of dogs in Sweden. Am. J. Vet. Res..

[52] Lechner E.S., Crawford P.C., Levy J.K., Edinboro C.H., Dubovi E.J., Caligiuri R. (2010). Prevalence of protective antibody titers for canine distemper virus and canine parvovirus in dogs entering a Florida animal shelter. J. Am. Vet. Med. Assoc..

[53] Taguchi M., Namikawa K., Maruo T., Orito K., Lynch J., Sahara H. (2011). Antibody titers for canine parvovirus type-2, canine distemper virus, and canine adenovirus type-1 in adult household dogs. Can. Vet. J..

[54] Ottiger H.P., Neimeier-Forster M., Stark K.D., Duchow K., Bruckner L. (2006). Serological responses of adult dogs to revaccination against distemper, parvovirus and rabies. Vet. Rec..

[55] Pawelec G., Larbi A. (2008). Immunity and ageing in man: Annual Review 2006/2007. Exp. Gerontol..

[56] Mansfield K.L., Burr P.D., Snodgrass D.R., Sayers R., Fooks A.R. (2004). Factors affecting the serological response of dogs and cats to rabies vaccination. Vet. Rec..

