# Supplementary material for: Prevalence of Neutralizing Antibodies to Canine Distemper Virus and Response to Vaccination in Client-Owned Adult Healthy Dogs
Source: Viruses. 2021 May 20;13(5):945. doi: 10.3390/v13050945 (PMC8160937; doi:10.3390/v13050945)
Supplement: Supplementary file 1 [file viruses-13-00945-s001.zip › viruses-1160051-supplementary.pdf]

viruses

MDPI

Supplementary

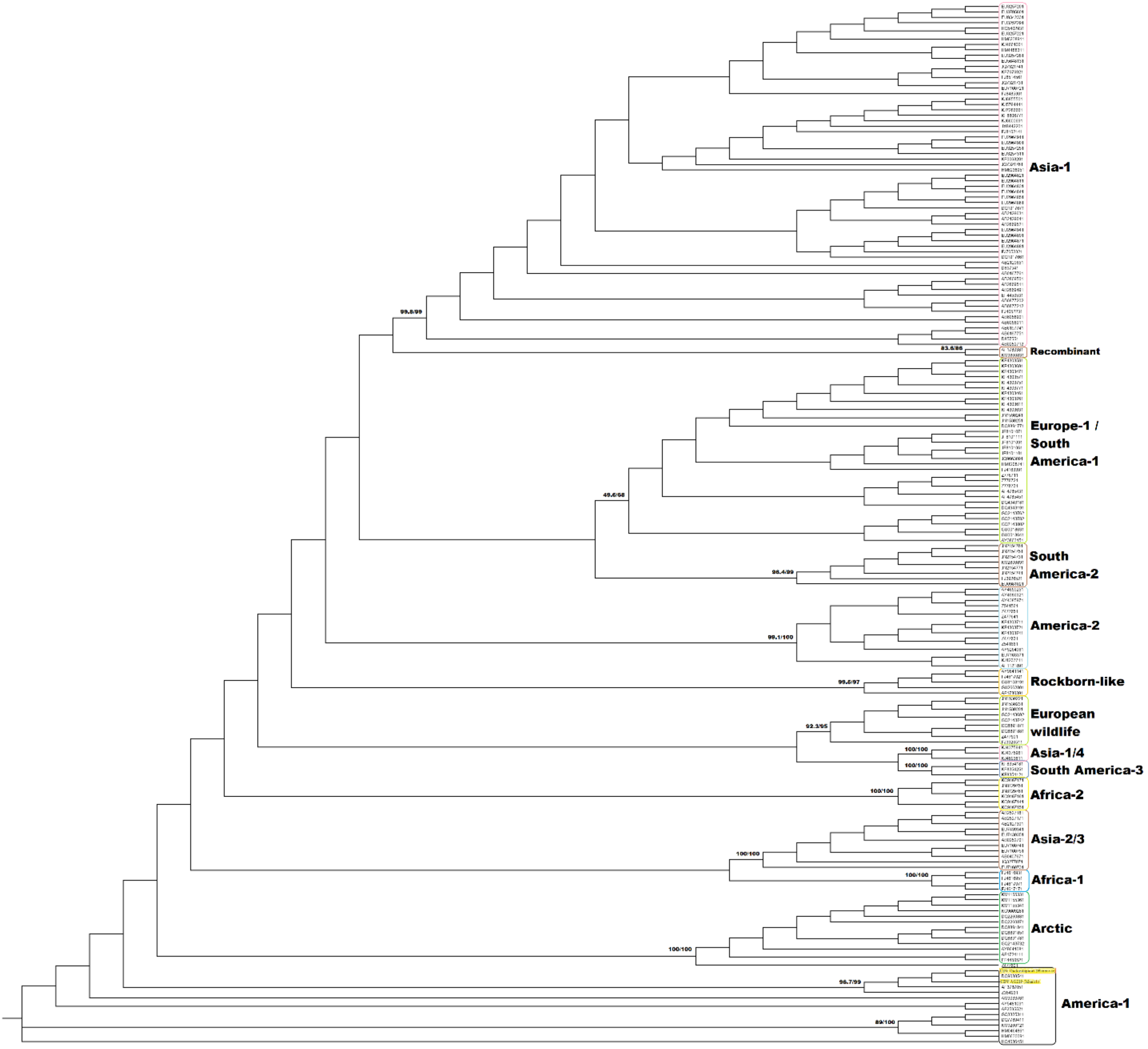

Figure 1. Phylogenetic position of test sequences (shaded yellow) among the clades of different lineages. Cluster of strains in each clade are color-bordered and denoted on the right. Clade credibility support values are denoted as bootstrap/posterior probability at key nodes.

Table S1. Anti-canine distemper antibody titers in virus neutralization using isolate AG219 in 97 dogs.

Dogs	Antibody Titer Against CDV on Day 0 (Before Vaccination)	Antibody Titer Against CDV on Day 7 (After Vaccination)	Antibody Titer Against CDV on Day 28 (After Vaccination)
1	160	320	160
2	40	40	40
3	<10	<10	<10
4	40	40	40

5	80	80	80
6	80	160	80
7	40	40	40
8	40	20	40
9	40	40	40
10	40	40	20
11	80	80	160
12	20	40	40
13	160	80	80
14	160	80	160
15	40	80	80
16	40	80	80
17	80	80	80
18	160	160	160
19	80	160	160
20	40	80	80
21	160	320	320
22	320	320	160
23	160	160	160
24	160	160	80
25	40	40	40
26	160	320	320
27	20	20	40
28	40	80	80
29	40	80	80
30	40	40	40
31	40	40	40
32	40	20	20
33	20	40	40
34	320	320	320
35	160	160	160
36	20	40	20
37	80	160	80
38	80	80	40
39	20	20	80
40	160	160	80
41	40	40	80
42	20	20	20
43	640	640	320
44	20	20	20
45	320	640	640
46	320	320	320
47	80	40	80
48	160	320	320
49	80	40	40
50	320	320	640
51	640	640	640
52	<10	<10	<10
53	10	20	20

54	160	80	160
55	80	80	160
56	160	160	80
57	<10	<10	<10
58	320	320	320
59	<10	<10	<10
60	640	640	640
61	80	80	160
62	80	160	80
63	80	160	80
64	80	160	160
65	320	320	160
66	40	40	40
67	320	640	320
68	80	80	80
69	80	80	160
70	20	20	20
71	160	160	160
72	640	320	640
73	40	80	40
74	40	40	40
75	20	40	40
76	80	80	80
77	10	10	10
78	40	40	40
79	40	20	20
80	40	40	40
81	40	20	40
82	20	20	20
83	20	40	20
84	40	40	20
85	20	10	10
86	80	40	40
87	20	40	20
88	80	160	160
89	20	20	10
90	<10	<10	<10
91	20	20	20
92	40	20	40
93	160	80	80
94	20	20	20
95	80	80	80
96	20	20	40
97	40	40	80
